# Evaluation of undiagnosed solitary lung nodules according to the probability of malignancy in the American College of Chest Physicians (ACCP) evidence-based clinical practice guidelines

**DOI:** 10.2478/raon-2013-0064

**Published:** 2014-01-22

**Authors:** Shinji Shinohara, Takeshi Hanagiri, Masaru Takenaka, Yasuhiro Chikaishi, Soich Oka, Hidehiko Shimokawa, Makoto Nakagawa, Hidetaka Uramoto, Tomoko So, Takatoshi Aoki, Fumihiro Tanaka

**Affiliations:** 1 Second Department of Surgery, School of Medicine, University of Occupational and Environmental Health, Kitakyushu 807, Japan; 2 Department of Radiology, School of Medicine, University of Occupational and Environmental Health, Kitakyushu 807, Japan

**Keywords:** inflammatory lung nodule, undiagnosed lung nodule, surgical resection, non-small cell lung cancer, solitary pulmonary nodules, computed tomography

## Abstract

**Background:**

This study retrospectively investigated the clinical significance of undiagnosed solitary lung nodules removed by surgical resection.

**Patients and methods:**

We retrospectively collected data on the age, smoking, cancer history, nodule size, location and spiculation of 241 patients who had nodules measuring 7 mm to 30 mm and a final diagnosis established by histopathology. We compared the final diagnosis of each patient with the probability of malignancy (POM) which was proposed by the American College of Chest Physicians (ACCP) guidelines.

**Results:**

Of the 241 patients, 203 patients were diagnosed to have a malignant lung tumor, while 38 patients were diagnosed with benign disease. There were significant differences in the patients with malignant and benign disease in terms of their age, smoking history, nodule size and spiculation. The mean value and the standard deviation of the POM in patients with malignant tumors were 51.7 + 26.1%, and that of patients with benign lesions was 34.6 + 26.7%. The area under the receiver operating characteristic (ROC) curve (AUC) was 0.67. The best cut-off value provided from the ROC curve was 22.6. When the cut-off value was set at 22.6, the sensitivity was 83%, specificity 52%, positive predictive value 90%, negative predictive value 36% and accuracy 77%, respectively.

**Conclusions:**

The clinical prediction model proposed in the ACCP guidelines showed unsatisfactory results in terms of the differential diagnosis between malignant disease and benign disease of solitary lung nodules in our study, because the specificity, negative predictive value and AUC were relatively low.

## Introduction

The recent prevalence of computed tomography (CT) scans in daily medical practice permits the identification of a large number of small, peripheral, undefined pulmonary lesions. The introduction of spiral CT has provided a technique with a high sensitivity for the detection of small lung cancers.^1^,^2^ The clinical characteristics of benign solitary pulmonary nodules, such as inflammatory pulmonary nodules, have not been fully investigated, and it is not always easy to distinguish between benign and malignant nodules using recent advanced radiographic modalities. Bronchoscopy under fluoroscopic guidance has come into wide use as a simple, safe and readily available sampling technique. However, the diagnostic yield of bronchoscopy for peripheral pulmonary lesions has been reported to be limited, because the identification of accessible bronchial routes to reach small peripheral pulmonary nodules is often difficult, and small peripheral pulmonary lesions may not be visible under fluoroscopic guidance.^3^ Furthermore, it is not always easy to obtain a sufficient amount of specimen to completely rule out malignancy. For these reasons, radiological evaluations, including observational CT, should be repeated.

Alternatively, surgical resection can be selected to decide on the course of treatment. The American College of Chest Physicians (ACCP) proposed evidence-based clinical practice guidelines based on a systematic literature review and discussion with a large multidisciplinary group of clinical experts and other stakeholders.^4^ This study retrospectively investigated the clinical significance of undiagnosed solitary pulmonary nodules (SPN) that were removed by surgical resection, and reviews whether using the probability of malignancy (POM) proposed in the ACCP evidence-based clinical practice guidelines is appropriate.

## Patients and methods

Pulmonary resections for lung nodules were performed in 759 patients between 2006 and 2010 in the Second Department of Surgery of the University of Occupational and Environmental Health. Among them, the clinic pathological data of 241 consecutive patients who underwent surgical resection to make a differential diagnosis of malignancy or non–malignancy were reviewed.

The preoperative assessments included chest radiography and CT of the chest, upper abdomen and brain. Whole lung CT scans were obtained with a 32-detector row CT scanner (Aquilion 32, Toshiba Medical Systems) or a 64-detector row CT scanner (Aquilion 64, Toshiba Medical Systems) using the following technique: 1 mm collimation, 0.5 second rotation time, 2 mm thick reconstructions, pitch (ratio of table travel per rotation to total beam width) of 27 or 53,120 kV. Automatic tube current modulation (z-axis modulation with Real E.C. technique, Toshiba Medical Solutions) was used with the noise level set at10 SD. Bronchoscopy was routinely performed to obtain a pathological diagnosis by a trans-bronchial lung biopsy. The patients’ records, including their clinical data, preoperative examination results, details of surgeries, and histopathological findings were also reviewed. Positron emission tomography (PET) scans were not routinely performed. We evaluated the probability of malignancy (POM) score for each undiagnosed solitary lung nodule according to validation of the Mayo Clinic Model on ACCP evidence-based Clinical Practice Guidelines.^5^,^6^ The Mayo Clinic Model used a multiple logistic regression analysis to identify the following six independent predictors of malignancy: older age; a history of smoking; a history of an extrathoracic cancer; larger nodule diameter; upper lobe location; and the presence of spiculation. The prediction model is described by the following equations:

Probability of malignancy=ex/(1+ex)

x=−0.6827+(0.0391×age)+(0.7917×smoke)+(1.3388×cancer)

+ (0.1274×diameter)+(1.0407×spiculation)+(0.7838×location)

where e is the base of the natural logarithm, age indicates the patient’s age in years; smoke indicates smoking history (1 = current or former smoker, 0 = never smoker); cancer indicates a history of an extrathoracic cancer 5 or more years before nodule identification (1 = yes, 0 = no or not specified); diameter indicates the largest nodule measurement in mm, reported on initial chest x-ray or CT; spiculation indicates mention of nodule spiculation on any imaging test report (1 = yes, 0 = no or not specified); and upper is the location of the nodule within the upper lobe of either lung (1 = yes, 0 = no). The ACCP evidence-based Clinical Practice Guidelines recommends that patients should be classified into the low risk category when the POM is less than 5%, moderate risk when the POM is 5 to 60 %, and high risk when the POM is more than 60%.^6^,^7^

A receiver operating characteristic (ROC) analysis (LABROC5 program by Metz *et al*., University of Chicago, IL, USA) was used to compare the observer performance in discriminating between malignant and benign cases. The accuracy of the detection was quantified by using the area under the ROC curve. The comparison between two groups was performed using Student’s t-test or the Mann–Whitney test. P values < 0.05 were considered to indicate a significant difference.

The investigators followed recommendations of the Helsinki Declaration.

## Results

The characteristics of the 241 patients are shown in Table 1. A total of 203 patients were diagnosed to have malignant lung tumors, while 38 patients were diagnosed with benign disease. In patients with malignant disease, 178 cases were diagnosed as primary lung cancer (143 cases of adenocarcinoma, 25 cases of squamous cell carcinoma, 5 cases of large cell carcinoma, 2 cases of small cell carcinoma, and 3 cases with other pathological types) and 25 cases were diagnosed as metastatic lung tumors. In the patients with benign disease, 13 cases were diagnosed as nonspecific inflammatory nodules, 11 cases as tuberculomas, 6 cases as hamarthomas, 4 cases as cryptococcosis, 3 cases as intrapulmonary lymph nodes and 1 case of atypical adenomatous hyperplasia. The mean age was 68.6 years in patients with malignant tumors and 65.3 years in the patients with benign disease, indicating that the patients with malignant tumors were significanty older than the patients with benign disease (p = 0.029). The former and current smokers were significantly more likely to be in the malignant tumor group (65% of subjects with malignant tumors) than to have benign disease (45% of subjects with benign disease) (p = 0.018).

Although 44% of patients with malignant tumors had malignancy in the histological evaluation of surgical specimen, 32% of patients with benign disease had malignancy in the postoperative histological evaluation. The mean tumor diameter was significantly larger in patients with malignant tumors (17.5 mm) than that of patients with benign disease (14.6 mm) (p = 0.025). Regarding spiculation, 122 malignant tumors (60%) showed spicules (55.2%), whereas 11 benign lesions (29%) had them. Malignant tumors had spicules significantly more frequently than did benign lesions (p < 0.001). No significant differences were observed in the patient gender, history of extra thoracic malignancy, or tumor location.

The mean value and the standard deviation of the POM in patients with malignant tumor were 51.7 + 26.1%, and that of patients with benign lesions were 34.6 + 26.7% (Table 2). According to the classification of the ACCP guidelines, the low risk group, moderate risk group and high risk group in patients with malignant tumors included 3 (1.5%), 112 (55.2%), and 88 (43.3%) patients, respectively. There were 4 patients (10.5%) who were at low risk, 25 (65.8%) at medium risk and 9 (23.7%) at high risk among the patients with benign lesions.

**FIGURE 1.**
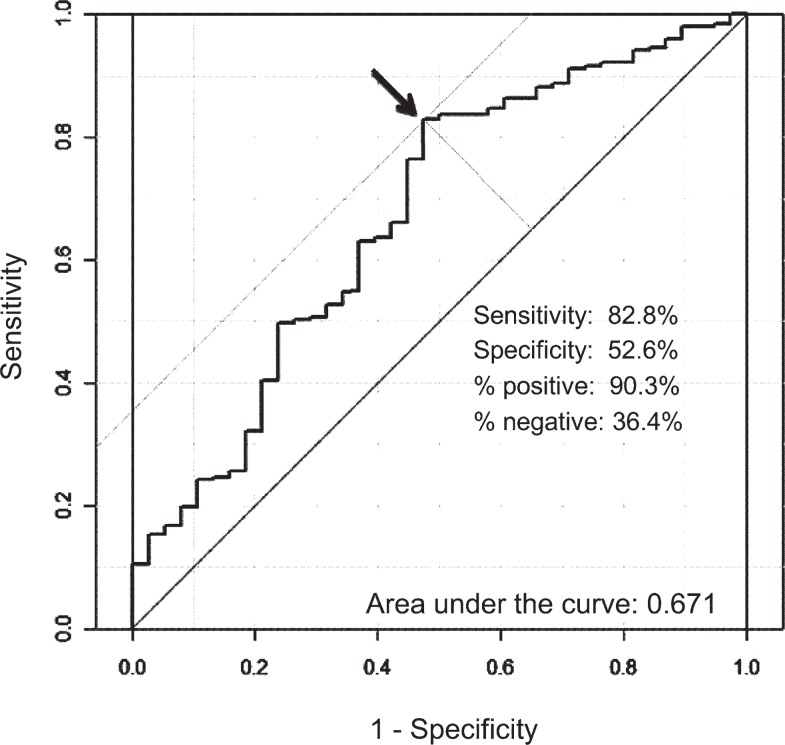
The Receiver Operating Characteristic Curve (ROC) for the prediction model of the ACCP guidelines. The area under the ROC curve was 0.67, and the best cut-off value provided from the ROC curve was 22.6.

The ROC curve of the POM is indicated in Figure 1. The area under the ROC curve (AUC) was 0.67. The best cut-off value provided from the ROC curve was 22.6. When the cut-off value was set at 22.6, the sensitivity, specificity, positive predictive value, negative predictive value and accuracy were 83%, 52%, 90%, 36% and 77%, respectively.

## Discussion

The incidental finding of a pulmonary nodule on CT is becoming an increasingly frequent event, and the management of these nodules has become an important issue.^1^,^8^ The most important concern is the differential diagnosis of benign disease from lung carcinoma. The optimal management of these SPN remains unclear. Minimizing the risk of unnecessary surgery should be considered, especially in patients with benign disease. However, it is not always easy to make a diagnosis preoperatively. The size of the nodule is a significant determinant of the diagnostic yield in bronchoscopy when evaluating lung nodules.^3^ The yield of bronchoscopy is particularly low for lesions ≤ 2 cm that are located in the outer third of the lung. In a recent review article, the sensitivity of conventional bronchoscopy for peripheral bronchogenic carcinoma < 20 mm was reported to be 34%, and was 63% for peripheral bronchogenic carcinoma > 20 mm.^4^

High resolution CT can evaluate the detailed characteristics of lung nodules, such as their size, morphology, and type of opacity. The risk for malignancy increases at a rate proportional to the diameter of the nodule.^9^ Solitary nodular shadows with pleural indentation and spicule formation are common radiological features of NSCLC. Wadahi *et al*. reported that the risk for malignancy in incidental or screening-detected nodules was approximately 20 to 30% in nodules with smooth edges; in nodules with irregular, lobulated, or spiculated borders, the rate of malignancy was higher, but varied across studies from 33 to 100%.^10^ In our study, there was statistically significant relationship between the tumor diameter and spiculation. Nodules that were pure ground-glass opacities (GGO), or predominately GGO were more likely to be malignant than solid nodules.^8^,^11^

FDG-PET scanning has been used to differentiate malignant solid lung nodules from benign nodules according to the basic concept that malignant pulmonary nodules have higher glucose metabolism. A recent review calculated the sensitivity and specificity of FDG-PET scanning to be 94.2% and 83.3%, respectively, for the identification of malignant pulmonary nodules.^5^,^10^ False-positive results are usually observed in lung nodules with an infectious or inflammatory etiology, such as due to tuberculosis, histoplasmosis, or rheumatoid nodules. Chun *et al*. reported that the maximum SUV uptake was significantly higher in patients with inflammation than in those with malignancy in part-solid nodules.^12^

In CT-guided percutaneous lung biopsy, the lesion size was also a determining factor for the diagnostic accuracy, and the sensitivity for malignancy was 76–88%.^13^,^14^ However, pneumothorax was the most common complication and occurred in 20–40% of patients.^15^ Tomiyama *et al.* reported severe complications in 9783 biopsies as follows; 0.061% with air embolism, 0.061% with tumor seeding at the site of the biopsy route, 0.10% with tension pneumothorax, 0.061% with severe pulmonary hemorrhage or hemoptysis, and 0.092% with haematothorax.^16^ The procedure is a safe and useful method, but it should be performed by appropriately trained and experienced physicians.^17^

After the use of a non-surgical approach such as bronchoscopy, or transthoracic needle aspiration/biopsy, the next step is to perform a radiological follow-up, since the evaluation of temporal changes in a small nodule may contribute to differentiating a malignant tumor from benign pathology.^18^,^19^ Alternatively, the diagnosis of a lung nodule may require surgical resection, after taking into account the benefits of a definitive diagnosis and treatment when compared with the surgical risk. Thoracoscopic resection is a minimally-invasive procedure for an undiagnosed solitary pulmonary nodule; it is useful for a definitive diagnosis not only to treat benign lesions, but also to plan the proper surgical procedure in case of malignancy.^20^,^21^ In patients with indeterminate lung nodules in the peripheral third of the lung, thoracoscopy should be recommended to perform a diagnostic wedge resection.^5^

There are several algorithms for the diagnostic prediction to manage solitary lung nodules.^9^,^22^,^23^ The Mayo Clinic model expressed the probability of malignancy using independent predictors of three clinical and three radiographic variables.^9^ Herder *et al*. reviewed and calculated the prediction of malignancy based on the Mayo Clinic model together with a PET scan.^23^ They demonstrated that the prediction model improved the AUC after the addition of the results of PET scans. Michael *et al*. identified the following four independent predictors of malignancy by using multivariate logistic regression analysis: positive smoking history; older age; larger nodule diameter; and quitting smoking.^24^ Each prediction model included the clinical and radiographic characteristics of lung cancer and seemed to provide good accuracy and calibration. In our study, a significant difference was found for the POM according to the calculation of the Mayo Clinic model, and the clinical prediction model was proven to have external validity. The AUC in our study was 0.67, which was low in comparison with those reported by other investigators (AUC 0.79 by Herder, AUC 0.83 by Swensen).

According to the ACCP guidelines, patients with a POM higher than 5% are classified into an intermediate–high risk group, and they are recommended to undergo additional tests, including PET, contrast-enhanced CT, transthoracic fine-needle aspiration biopsy or bronchoscopic biopsy, or video-assisted thoracoscopic surgery. The clinical prediction model proposed in the ACCP guidelines is an overestimated model, with few patients being categorized into the low risk group in the present study. While the POM overestimation model might result in unnecessary surgery or biopsy in some patients with benign nodules (a false positive diagnosis), underestimation models might lead to a delayed diagnosis and missed opportunities for a surgical cure in patients with malignant nodules (a false negative diagnosis). Schultz *et al*. proposed that clinicians should be mindful of the prevalence of malignant nodules in their practice setting.^25^

In the present study, we retrospectively reviewed the clinic pathological data of patients who had nodules measuring 7 mm to 30 mm and a final diagnosis established by histopathology. Therefore, the effect of “work up bias” should be considered in the evaluation of the prediction model.^26^ Patients with a positive test result are likely to undergo a procedure for tissue pathologic verification, resulting in a disproportionately large share of patients undergoing verification having a positive test. For this reason, sensitivity (positive test when disease is present) appears to be high. Although the specificity, negative predictive value, and AUC were relatively low in our study, the clinical prediction model proposed in the ACCP guidelines have validity to prevent a false negative diagnosis. We consider that this model should not be used as a stand alone test, but that the model can help to adjust the diagnostic work-up. Further investigations should be necessary to evaluate the benefits of the clinical prediction model of ACCP guidelines in various cohorts as follows; all population having a mass screening CT, or high risk groups such as elderly, smokers or patients with history of cancer.

## Figures and Tables

**TABLE 1.** The characteristics of 241 patients who underwent surgical resection for an undiagnosed solitary lung nodule

	**Malignant tumor (n = 203)**	**Benign disease (n = 38)**	**P**
Mean age	68.6	65.3	0.029
Male (%)	122 (60)	25 (66)	0.509
Smoker (%)	132 (65)	17 (45)	0.018
Past history of cancer (%)	90 (44)	12 (32)	0.144
Mean tumor diameter (mm)	17.5	14.6	0.025
Spicula (%)	122 (60)	11 (29)	<0.001
Tumor in upper lobe (%)	66 (33)	18 (47)	0.078

**TABLE 2.** The comparison of the probability of malignancy between malignant tumor and benign disease

	**Malignant tumor (n = 203)**	**Benign disease (n = 38)**	**P**
Mean probability of malignancy	51.7	34.6	p < 0.001
Low risk group (%)	3 (1)	4 (10)	
Median risk group (%)	112 (55)	25 (66)	
High risk group (%)	88 (43)	9 (24)	
